# Systemic Treatment for Advanced and Metastatic Malignant Peripheral Nerve Sheath Tumors—A Sarcoma Reference Center Experience

**DOI:** 10.3390/jcm9103157

**Published:** 2020-09-29

**Authors:** Paweł Sobczuk, Paweł Teterycz, Anna M. Czarnecka, Tomasz Świtaj, Hanna Koseła-Paterczyk, Katarzyna Kozak, Sławomir Falkowski, Piotr Rutkowski

**Affiliations:** 1 Department of Soft Tissue/Bone Sarcoma and Melanoma, Maria Sklodowska-Curie National Research Institute of Oncology, 02-781 Warsaw, Poland; pawel.teterycz@pib-nio.pl (P.T.); am.czarnecka@pib-nio.pl (A.M.C.); tomasz.switaj@pib-nio.pl (T.Ś.); hanna.kosela-paterczyk@pib-nio.pl (H.K.-P.); katarzyna.kozak@pib-nio.pl (K.K.); Slawomir.falkowski@pib-nio.pl (S.F.); piotr.rutkowski@pib-nio.pl (P.R.); 2 Department of Experimental and Clinical Physiology, Laboratory of Centre for Preclinical Research, Medical University of Warsaw, 02-097 Warsaw, Poland; 3 Department of Experimental Pharmacology, Mossakowski Medical Research Centre, Polish Academy of Sciences, 02-106 Warsaw, Poland

**Keywords:** MPNST, sarcoma, chemotherapy, doxorubicin, ifosfamide, pazopanib

## Abstract

Malignant peripheral nerve sheath tumor (MPNST) is a rare type of soft tissue sarcomas. The localized disease is usually treated with surgery along with perioperative chemo- or radiotherapy. However, up to 70% of patients can develop distant metastases. The study aimed to evaluate the modes and outcomes of systemic treatment of patients with diagnosed MPNST treated in a reference center. In total, 115 patients (56 female and 59 male) diagnosed with MPNST and treated due to unresectable or metastatic disease during 2000–2019 were included in the retrospective analysis. Schemes of systemic therapy and the outcomes—progression-free survival (PFS) and overall survival (OS)—were evaluated. The median PFS in the first line was 3.9 months (95% CI 2.5–5.4). Doxorubicin-based regimens were the most commonly used in the first line (50.4% of patients). There were no significant differences in PFS between chemotherapy regimens most commonly used in the first line (*p* = 0.111). The median OS was 15.0 months (95% CI 11.0–19.0) and the one-year OS rate was 63%. MPNST are resistant to the majority of systemic therapies, resulting in poor survival in advanced settings. Chemotherapy with doxorubicin and ifosfamide is associated with the best response and longest PFS. Future studies and the development of novel treatment options are necessary for the improvement of treatment outcomes.

## 1. Introduction

Recent years have brought a significant improvement in the field of systemic therapy in oncology. Targeted therapies and immunotherapy have revolutionized the treatment of various cancers at the metastatic stage. However, the treatment of many tumors is still challenging, based mainly on classic chemotherapy and associated with poor outcomes. Soft tissue sarcomas (STS) are one of such cancers.

STS is a diverse group of rare tumors with an estimated incidence of 4–6 cases per 100,000 a year [1]. Currently, over 100 STS subtypes have been characterized and this number is continuously growing [2]. One of the STS subtypes is a malignant peripheral nerve sheath tumor (MPNST), a highly-aggressive sarcoma that arises from neuroectodermal cells of peripheral or cranial nerves. It accounts for approximately 4–5% of all STS, which result in approximately 1500 new cases a year in the European Union [3,4]. The risk of development of MPNST is over 4000 times higher in patients with type 1 neurofibromatosis (NF1; von Recklinghausen’s syndrome); however, in more than 50%, it is a sporadic disease. It can also occur in a previously irradiated field [5].

Surgery is a gold standard for the treatment of localized disease and is often combined with perioperative radio- and/or chemotherapy for high-risk tumors. Even with optimal radical surgery with negative margins, local recurrence and distant metastases are observed in the course of the disease. Metastases are found in 40–70% of patients, usually in the lungs, liver, or bones [6].

Treatment of metastatic disease is highly challenging due to a lack of high-quality evidence on the efficacy of systemic treatments in this sarcoma type. Anthracycline-based chemotherapy is most commonly used as the first-line treatment. A combination of doxorubicin with ifosfamide has been shown to be associated with the best outcomes and is generally recommended in this situation [7]. The evidence for other regimens is very limited. Due to the rarity of MPNST, conducting large randomized trials is almost impossible. Various small targeted molecules were tested in small cohorts of MPNST patients, but trials have not brought any progress in the treatment of this disease [8]. The survival of patients with metastatic or unresectable disease is poor, with a median progression-free survival of approximately four months and overall-survival of 13 months [9].

This retrospective study aimed to analyze the treatment modalities and outcomes of patients with advanced, unresectable, or metastatic MPNST receiving systemic therapy in the reference center. Moreover, we attempted to identify prognostic factors associated with progression-free survival and overall survival in this cohort.

## 2. Materials and Methods

### 2.1. Patients Selection and Data Collection

We reviewed the electronic health records of patients hospitalized in Maria Sklodowska-Curie National Research Institute of Oncology in Warsaw, Poland, who started treatment for metastatic/unresectable MPNST between January 2000 and June 2019. Patients with a diagnosis of MPNST, confirmed by pathologists experienced in soft tissue sarcoma, who received at least one line of systemic treatment because of unresectable or metastatic disease, were selected. The disease was considered unresectable when the tumor was located in a difficult anatomical location, infiltrating vital structures, or if the patient was not feasible for surgery. The decision about unrespectability was made by the multidisciplinary tumor board. Patients with incomplete data for the date of diagnosis and treatment of primary tumor and unwilling to undergo treatment in our institution were excluded. 

Patients’ demographic and clinicopathological data—sex, age at the diagnosis, age at the start of systemic treatment, NF1 status, primary tumor size, location, grade, treatment of primary tumor, and location of metastases—were collected. Furthermore, we retrieved data on the type of systemic treatment—regimens, doses, and tumor responses. Imaging was performed every 2–3 months and additional scans could be performed if clinically indicated. In the case of suspicion of clinical progression, patients underwent imaging to confirm progression, unless no other therapy could be proposed to the patient. Tumor responses were assessed based on the available imaging reports and electronic health records and evaluated according to the RECiST 1.0 [10] or 1.1 criteria [11], depending on the time of treatment. Images were not re-reviewed. 

### 2.2. Statistical Analyses

Patients were followed for survival status and disease recurrence in our out-patient clinic. Progression-free survival (PFS) was calculated from the start of the treatment up to the date of radiologically confirmed or clinical progression or last follow-up (censored). Overall survival (OS) was calculated from the date of the start of the treatment up to the date of death or last follow up (censored). The exact dates of death in patients lost to follow up were received from the Polish National Cancer Registry. The patients alive or without disease progression at the date of data retrieval were censored (30 May 2020).

Discrete variables were summarized as numbers and percentages while continuous ones with mean and range in the case of a normal distribution or with median and interquartile range when distribution was skewed. The Chi-squared test was used for between-group comparisons. Kaplan–Meier estimator with the log-rank test was used for plotting and assessing differences between survival curves. With all point estimates, 95% confidence intervals (CI) were reported. No adjustment for multiple testing was performed. All analyses and figures drawing were performed using IBM SPSS Statistics for Windows version 26 (IBM Corp). The differences were considered statistically significant if the *p* values were <0.05. 

## 3. Results

### 3.1. Patients Characteristics

In total, 115 patients (56 female and 59 male) were included in the study. The median age at the time of diagnosis of the primary tumor was 43.1 years (range 18–82). Twenty-three patients (20.0%) had NF-1 related MPNST and 92 (80.0%) sporadic tumors. Overall, 8.7% (*n* = 10) patients presented with distant metastases and 1.7% (*n* = 2) with lymph node metastases at the diagnosis of the primary tumor. 

The primary tumors were most often located in the proximal part of lower extremities in 31.3% of all cases, followed by the trunk 18.3%, distal part of the lower extremity in 13.9%, upper extremity in 13.9%, and retroperitoneal in 11.3%. Epithelioid subtype was present in 1.7%, Malignant Triton tumor histology in 3.5% of cases, and the remaining 94.8% had not-otherwise specified. Most of the tumors were Grade 3 or unknown (69.6%), with Grade 2 in 26.1% and Grade 1 in 4.3%. The median primary tumor size was 12 cm. Overall, 84.3% (*n* = 97) of patients underwent previous surgery with curative intent and 22.6% (*n* = 26) patients received previous perioperative chemotherapy. 

The median age at the time of the start of the systemic treatment was 44 years (range 18–82). At the time of the start of palliative systemic treatment, 50.4% of patients had local recurrence or unresected primary tumor, 62.6% developed metastases to the lungs, 12.2% to the lymph nodes, 6.1% to the bones, and 5.2% to the liver. Other locations of metastases included muscles, pancreas, spleen, and visceral metastases. Clinicopathological characteristics of patients are described in Table 1.

### 3.2. Treatment Modalities and Outcomes

Median follow up was 11.8 (1–147) months. In total, 115 patients received at least one line of therapy, 74 patients two lines, 40 patients three lines, and 21 patients received four or more lines. One patient received eight lines of systemic therapy. Details of chemotherapy regimens are provided in Table A1.

#### 3.2.1. First-Line Treatment

Twenty-five (21.7%) patients were treated in the first line due to localized, unresectable disease, and 90 (78.3%) because of distant metastases with or without local recurrence/primary tumor. In the first line, doxorubicin-based regimens were the most commonly used in 50.4% of patients, followed by high-dose ifosfamide in 34.8% and etoposide with ifosfamide in 14.8%. Among anthracycline-based regimens, doxorubicin was most often combined with dacarbazine (ADIC regimen). The selection of chemotherapy differed significantly between patients who receive and did not receive perioperative chemotherapy (*p* = 0.025). ADIC regimen was most common in previously treated patients and high-dose ifosfamide in the untreated population (Table A2). Combinations of chemotherapy changed with time, depending on the year of treatment initiation (*p* < 0.001; Table A2). 

Besides chemotherapy, some patients underwent resection of the recurrence, metastasectomy, or were additionally treated with radiotherapy. In the first line of palliative, surgery was performed in 20 (17.4%) patients, and 15 (13.0%) patients were treated with radiotherapy. Sixteen percent of patients with unresectable localized disease (*n* = 4) received radiotherapy.

Imaging data and assessment of tumor response were available for all patients in the first line. The objective response rate to chemotherapy in the first line was 25.2% (*n* = 29) with 1 complete response (in patients receiving doxorubicin and ifosfamide) and 28 partial responses. Forty-one patients had stable disease resulting in a clinical benefit rate of 60.9%. During the follow-up, 107 (93.0%) patients have experienced disease progression. Patients discontinued the therapy due to progression during the treatment or after follow-up if the maximal number of cycles was already administered. We did not observe any toxicities leading to treatment discontinuation or treatment-related deaths.

**Figure 1:**
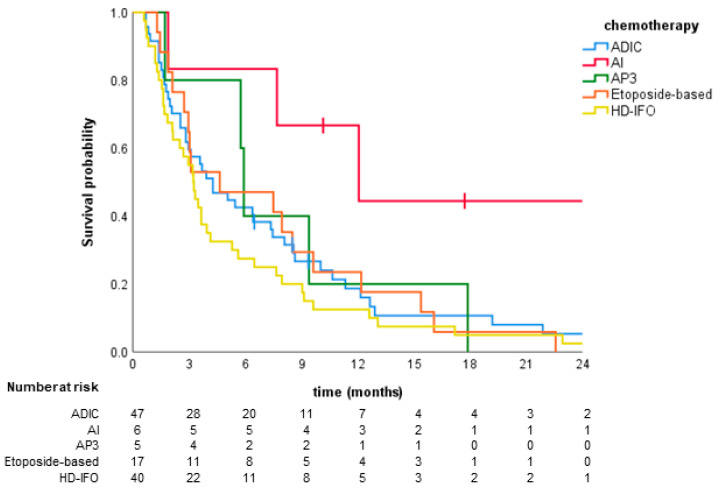
Progression-free survival stratified by chemotherapy regimens most commonly used in the first line. ADIC, doxorubicin + dacarbazine; AI, doxorubicin + ifosfamide; AP3, doxorubicin + cisplatin; EI, etoposide + ifosfamide; HD-IFO, high-dose ifosfamide.

The median PFS in the first line was 3.9 months (95% CI 2.5–5.4). One-year PFS rate was 23%. There were no significant differences in PFS between chemotherapy regimens most commonly used in the first line (*p* = 0.111) (Figure 1); however, patients receiving doxorubicin with ifosfamide had numerically longer PFS than other patients (Table 2). In the univariate logistic regression model, doxorubicin + ifosfamide regimen was associated with a lower risk of progression compared to doxorubicin + dacarbazine (HR 0.30; 95% CI 0.09–0.96; *p* = 0.042) (Table 2).

Patients with NF1-related MPNST had shorter median PFS in first-line therapy, 2.7 vs. 3.9 months, respectively, but the difference was not statistically significant (*p* = 0.14). Patients who received additional palliative local therapy had significantly longer median PFS—8.0 vs. 3.6 months (*p* = 0.044) in patients treated with surgery and 9.1 vs. 3.3 months (*p* = 0.041) in those receiving radiotherapy.

There were no differences in median PFS depending on sex (*p* = 0.382), age (*p* = 0.279), tumor grade (*p* = 0.290), the disease (local or metastatic, *p* = 0.261), previous surgery for primary tumor (*p* = 0.638), or previous chemotherapy use (*p* = 0.335). There was no significant difference between patients with unresectable primary tumors and patients with metastatic disease (*p* = 0.307).

Additional explorative analysis was performed to compare PFS in a cohort of patients who received chemotherapy alone (*n* = 82) or together with surgery or radiotherapy (*n* = 33). The median PFS in the first-line treated with chemotherapy alone was 3.1 months (95% CI 2.5–3.7), and in patients who were feasible for and received concurrent surgery or radiotherapy it was 8.6 months (95% CI 7.1–10.1). There were no significant differences in PFS among the chemotherapy regimens most commonly used in the first line in both settings (*p* = 0.118) (Table 3).

#### 3.2.2. Second and Further Lines 

The most commonly used systemic therapies in the second and third line of treatment were doxorubicin-based schemes, followed by gemcitabine-based chemotherapy. A summary of chemotherapy regimens used in second and further lines of therapy is presented in Table 4. 

**Figure A1:**
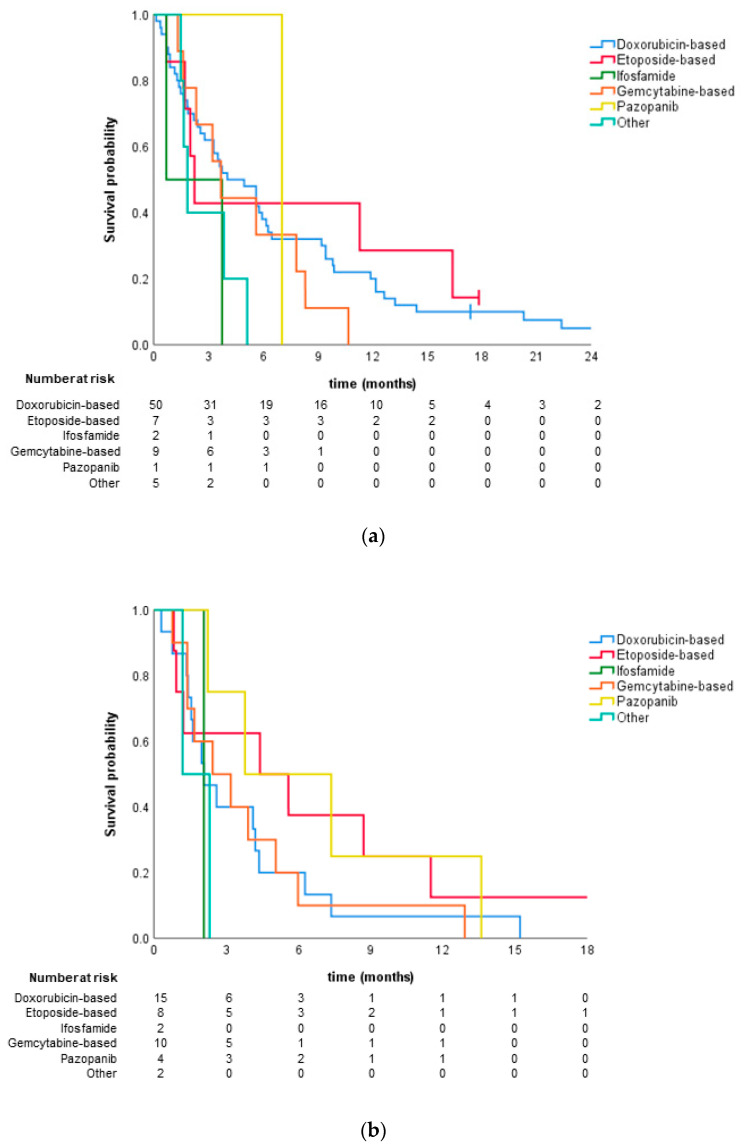
Progression-free survival stratified by chemotherapy regimens used: in the second line of treatment (**a**); and third line of treatment (**b**).

The median PFS was 3.7 months (95% CI 2.0–5.5) in the second and 3.2 (95% CI 2.3–5.6) in the third line. There were no differences in PFS between different regimens used in the second and third lines. There were no significant differences between regimens used in the second (*p* = 0.309) and third lines of treatment (*p* = 0.411) (Figure A1).

In the second line of treatment, surgery was performed in 11 (14.9%) and was associated with longer median PFS than in not operated patients, 10.7 vs. 3.3 months (*p* = 0.001). There was no benefit in patients receiving radiotherapy (*n* = 9; 12.2%; *p* = 0.259).

#### 3.2.3. Analysis of Systemic Therapy Regimens Irrespective of the Line of Treatment

**Figure 2:**
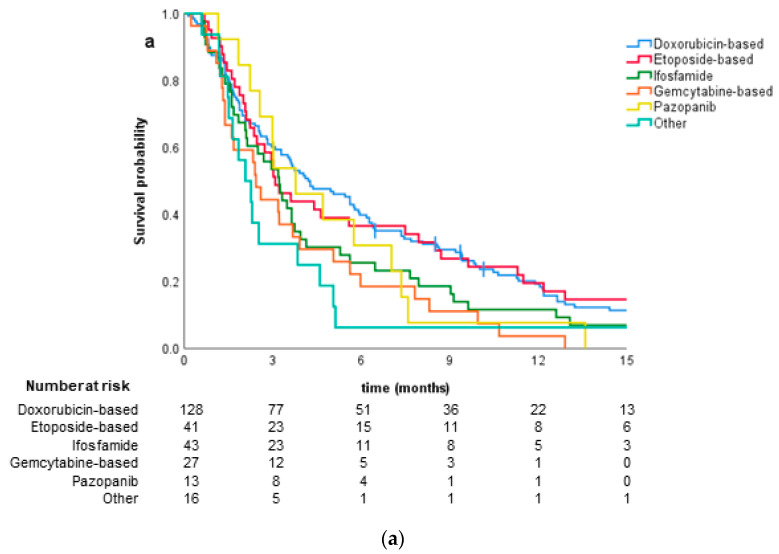

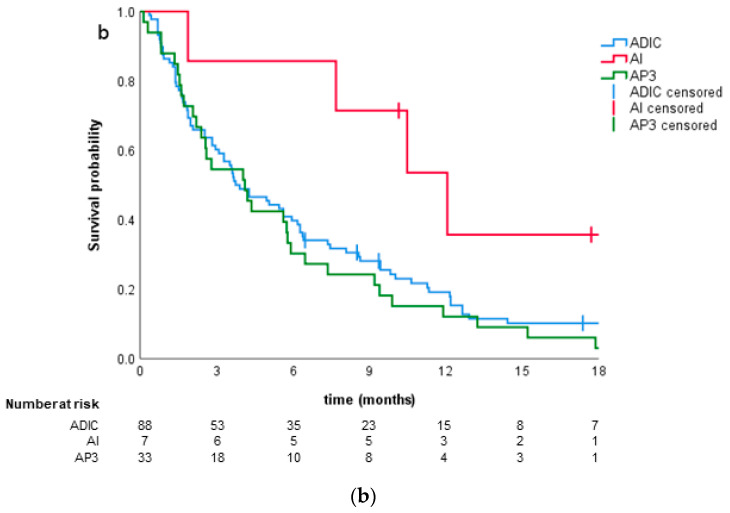
Comparison of progression-free survival: for all systemic therapy groups irrespective of the line of treatment (**a**); and for various doxorubicin-based regimens (**b**). ADIC, doxorubicin + dacarbazine; AI, doxorubicin + ifosfamide; AP3, doxorubicin + cisplatin.

We performed an exploratory analysis of all systemic therapy regimens, irrespective of the line in which they were used. In total, 269 therapies were administered: 128 (47.6%) were doxorubicin-based, 42 (15.6%) etoposide-based, 43 (16.0%) were high-dose ifosfamide, 27 (10.0%) gemcitabine-based, 13 (4.8%) included pazopanib, and 16 (5.09%) other drugs. Twenty-seven (23.5%) patients received two or more doxorubicin-based regimens. There were significant differences in median PFS between regimens with the longest in doxorubicin-based regimens—4.2 (95% CI 2.8–5.9) months—and pazopanib—3.8 months (95% CI 1.8–5.8) (Figure 2a). Use of gemcitabine-based regimens was associated with significantly worse prognosis compared to doxorubicin-based regimens with HR 1.74 (95% CI 1.14–2.66, *p* = 0.01) (Table A3).

Among doxorubicin-based regimens, doxorubicin with ifosfamide was associated with better PFS compared to combination with dacarbazine or cisplatin, 12.1 vs. 3.75 vs. 4.1 months (*p* = 0.041) (Figure 2b). Median PFS for anthracycline-based chemotherapy in the first line was 5.5 months compared to 3.65 months in further lines, but the difference was not significant (*p* = 0.111).

We performed an exploratory analysis of all systemic therapy regimens, irrespective of the line in which they were used, excluding patients who received local therapy (radiotherapy/surgery). In total, 200 therapies were administered: 92 (47.6%) were doxorubicin-based, 36 (15.6%) etoposide-based, 28 (16.0%) were high-dose ifosfamide, 20 (10.0%) gemcitabine-based, 12 (4.8%) included pazopanib, and 12 (5.09%) other drugs. There were no significant differences in median PFS between regimens: doxorubicin-based regimens—3.1 (95% CI 1.9–4.2) months—and pazopanib—3.0 (95% CI 1.7–4.4) months. Use of gemcitabine-based regimens was associated with significantly worse prognosis comparing to doxorubicin-based regimens with HR 1.83 (95% CI 1.12–3.00, *p* = 0.016) (Table A4). Among doxorubicin-based regimens, there were no differences in PFS when comparing the combination of doxorubicin and dacarbazine with doxorubicin and cisplatin, 3.1 (95% CI 2.0–4.2) vs. 2.6 (95% CI 0.2–4.9) months (*p* = 0.908). Doxorubicin + ifosfamide was excluded from the analysis due to the low number of patents (*n* = 2). Median PFS for anthracycline-based chemotherapy in the first line was 3.9 months compared to 2.4 in further lines, but the difference was not significant (*p* = 0.143).

### 3.3. Overall Survival

The median OS in patients treated with palliative intent was 15.0 (95% CI 11.0–19.0) months. One-year OS rate was 63%. Patients with NF1-related MPNST had shorter median OS, 11.2 vs. 16.8 months, respectively, but the difference was not statistically significant (*p* = 0.171). There were no significant differences in OS when stratified by chemotherapy regimens most commonly used in the first line (*p* = 0.2) or by use of perioperative chemotherapy (*p* = 0.7). There was no significant difference between patients with unresectable primary tumor and patients with metastatic disease (*p* = 0.792).

The median OS was different depending on the number of lines of palliative treatment received by the patients: 15.0 (95% CI: 6.9–23.1) months in patients who received only one line, 12.1 (95% CI: 10.4–13.8) months with two lines, 12.1 (95% CI: 8.3–15.8) months with three lines, and 33.6 (95% CI: 23.9–43.2) months with four or more lines. However, this was not statistically significant (*p* = 0.084). 

## 4. Discussion

Surgery is the gold standard of treatment for localized MPNST; however, a significant proportion of patients develop local recurrence or distant metastases even after radical resection. In the advanced settings, curative treatment is usually impossible and palliative systemic therapy remains the main modality used to improve patients survival and quality of life. This is a challenging situation due to a lack of high-quality data on the use of chemotherapy in palliative treatment. Current practice in terms of first-line therapy of advanced MPNST has been shaped by the pooled analyses of 12 nonrandomized and randomized European Organization for Research and Treatment of Cancer (EORTC) Soft Tissue and Bone Sarcoma Group trials, including 175 patients, which showed superiority of anthracycline-based chemotherapy, especially doxorubicin + ifosfamide regimen [7]. Most of the other data come from retrospective analyses or the few clinical trials conducted in patients with a variety of STS, and only a few patients with MPNST. Here, we present treatment outcomes in one of the largest cohort of patients with advanced or metastatic MPNST. 

MPNST is considered to be highly chemotherapy-resistant, which is reflected in poor survival. Median PFS in the first line of palliative treatment in our cohort was 3.9 months and one-year PFS was 23%, which is in line with previous observations from a French group that reported mPFS of 4.3 months and one-year PFS of 21.6% [9]. Median PFS was shorter in the subgroup of patients treated only with systemic therapies comparing to those patients who received additional radiotherapy or underwent surgical resection of metastases or recurring tumor. This shed light on the impact of local treatment in patients with advanced MPNST. 

Half of the patients in the first line received doxorubicin-based chemotherapy. The choice of chemotherapy has evolved during the analyzed period (Table A3), with a significant proportion of patients receiving high-dose ifosfamide before 2010, and increased frequency of doxorubicin- and etoposide-based regimens in the recent years, which reflects changes in the understanding of MPNST biology and reports on the efficacy of available therapies [12]. 

In terms of PFS in the first line in the whole study population, doxorubicin with ifosfamide was the most effective regimen with a 70% reduction in the risk of progression, compared to doxorubicin with dacarbazine. It has not been confirmed when patients who received additional local treatment were excluded. These observations have to been interpreted with caution due to the very low number of patients receiving doxorubicin with ifosfamide (six patients in the first line).

The exploratory analyses of all administered therapies, irrespective of the line, also confirmed the superiority of doxorubicin-based schemes. Thus, we confirm previously reported observation regarding the use of doxorubicin as the most effective treatment in the first line [7,9,13,14,15]. As already reported in the metanalyses of 12 EORTC studies, the addition of ifosfamide to doxorubicin is associated with longer PFS comparing to doxorubicin monotherapy (26.9 vs. 17 weeks). Our results regarding this combination must be interpreted with caution due to the low number of patients receiving it. Generally, doxorubicin with ifosfamide is associated with a higher number of side effects [13,16], compared to doxorubicin alone or with dacarbazine, thus is usually used in fit patients with better performance status and fewer comorbidities, especially with lower risk of cardiovascular complications. It is important to underline that some patients in our cohort received more than one doxorubicin-based regimen despite previous progression when treated with doxorubicin. Reintroduction of such schemes was considered individually for each patient, especially if a response to doxorubicin was observed previously. The efficacy of further doxorubicin-based regimens was much lower compared to the first usage. We observed some activity of etoposide-based regimens, especially in the first line, where median PFS was similar to in patients receiving doxorubicin with dacarbazine. Efficacy of etoposide in the MPNST was reported in clinical studies [17,18], case reports [19], and MPNST cell lines [20,21]. Etoposide in combination with ifosfamide was also used as histology-tailored neoadjuvant chemotherapy when compared with standard doxorubicin–ifosfamide regimen; however, it did not show any superiority [22,23]. 

In addition, a tyrosine kinase inhibitor pazopanib has shown some activity in MPNST when used in further lines of treatment; however, with a median PFS of 3.8 months, its activity is moderate. In our cohort, pazopanib was used mostly in the third or fourth line, which could cause shorter PFS than in previous reports. In a small phase II trial conducted in 12 patients, median PFS was 5.4 months with a clinical benefit rate of 50% at 12 weeks [24], while, in a small retrospective series of five cases, PFS was 6.5 months [24]. 

The shortest PFS in the first line was reported in patients treated with ifosfamide, similarly to in the metanalysis of EORT clinical trials [7]. Overall, irrespective of the line, the shortest PFS was observed when gemcitabine-based regimens were used. Preclinical [21,25] and clinical data suggest that gemcitabine can be effective in some cases. This discrepancy can be caused by the use of gemcitabine-based chemotherapy most commonly in further lines of treatment (third line and further) when the disease is more extensive and patients can be in the worse condition after previous therapy. There are data suggesting that treatment outcomes in patients with NF-1 associated MPNST are worse than in patients with sporadic-disease, however it is associated with a lot of controversies. Valentin et al. [9] reported shorter PFS and OS, suggesting lower chemosensitivity of NF-1-related tumors; however, we did not find such significant differences. In our cohort of patients, PFS was only slightly shorter, with no statistical significance. Moreover, we did not find differences in the overall survival. We have to underline that the proportion of patients with NF-1-related MPNST in our population was lower than in previous studies, probably due to retrospective nature and underreporting, thus findings regarding this subgroup have to be interpreted with caution. 

Importantly, palliative radiotherapy or surgery used along with systemic therapy significantly improved outcomes. It seems that the use of those modalities in patients with locally-advanced or oligometastatic disease and good response to chemotherapy can improve patients’ survival. We did not find differences in treatment outcomes based on age, sex, extent of the disease, location of primary tumor, tumor grade, or size.

Assessment of toxicity was not the aim of this study, but we did not observe treatment-related deaths in our cohort. Treatment toxicity and quality of life have to always be considered when selecting the treatment, especially in patients with relatively short survival, as in MPNST. Doxorubicin with ifosfamide is more toxic than doxorubicin alone or doxorubicin with ifosfamide, which can limit the use of this regimen in palliative settings [13]. 

Median OS was 15 months and one-year OS rate 63%, which is slightly better than in the French cohort [9]. Interestingly, we observed longer OS in patients receiving four or more lines of palliative treatment. Except for the higher proportion of patients who underwent resection of the primary tumor, we did not find any differences between patients with four and more lines and patients with up to three lines. This may suggest that, even with limited effect and short PFS on each line of therapy, chemotherapy can be beneficial in terms of survival and should be considered in patients with good performance status. 

Despite reporting on one of the largest cohort of patients with MPNST treated with systemic therapies, our study possesses several limitations. The key ones are the retrospective nature and long period when patients were treated. With new published data and our own experience, choice of chemotherapy regimens was changing and this could affect the results (Table A1). Additionally, the number of patients with NF1-related MPNST can be underestimated due to underreporting or underdiagnosis of neurofibromatosis.

Moreover, measures used to assess the efficacy of treatment can lead to some bias. In this study, we focused on the reporting of survival outcome—PFS. PFS strongly depends on the frequency of imaging and if scans are done less frequently it can result in the improvement of PFS in real-world data reports compared to clinical trials. In our study, scans were performed every 2–3 months, which. is a standard interval in routine clinical practice and clinical trials; however, in MPNST patients with survival of approximately three months, the frequency of imaging could significantly affect the PFS. On the other hand, the response rate is another widely used endpoint for assessment of treatment efficacy. In our study, the objective response rate in first line was 25.2%, with the highest in patients treated with doxorubicin and ifosfamide (66.7%); however, the data can be biased due to retrospective nature and small number of patients. Considering the fact that MPNST is a rather chemotherapy-resistant malignancy, relying only on objective response rate can be difficult because only a minority of patients will experience disease regression. Achieving disease stabilization, which results in longer PFS, can be a measure of treatment efficacy. The biology of MPNST is still not completely understood; thus, differences in survival and responses to different therapies may reflect the natural course of the disease rather than the efficacy of therapy. Due to the rarity of the disease, it is not possible to recognize different subgroups among MPNST patients and more research is necessary.

In light of the current knowledge and results of our study, doxorubicin-based chemotherapy remains the standard of care in the front line of treatment for advanced or metastatic MPNST. To achieve the best outcomes, the combination with ifosfamide seems the most promising, especially for patients in whom higher toxicity can be accepted. More challenging is a selection of treatment for patients who progressed during doxorubicin treatment, have contraindications for such regimen, or already received a lifetime cumulative dose of anthracyclines. In those cases, etoposide-based chemotherapy could be considered. In addition, pazopanib can be used in the palliative setting. Importantly, the quality of evidence for systemic therapies choice, besides anthracyclines, is low.

Overall, available therapies offer very limited benefits to patients with advanced MPNST. There is a high need for new options, preferably targeted therapies, however many already completed trials have failed to show any benefit of new compounds [8]. Several clinical trials are ongoing (NCT02691026, NCT02180867, NCT02584647, NCT02584647, NCT02700230, and NCT03880123) and their results are awaited in the following years. 

## Figures and Tables

**Table 1:** Clinicopathological characteristics of patients.

Factor		Overall Population (*n* = 115) *n* (%)
Gender	female	56 (48.7%)
	male	59 (51.3%)
Age at first diagnosis	median (range)	43.1 (18–82)
Age at start of systemic treatment	median (range)	44 (18–84)
Location of primary tumor	Arm	16 (13.9%)
	Lower Distal	16 (13.9%)
	Trunk	21 (18.3%)
	Head and Neck	7 (6.1%)
	Visceral	6 (5.2%)
	Lower Proximal	36 (31.3%)
	Retroperitoneal	13 (11.3%)
Grade	G1	5 (4.3%)
	G2	30 (26.1%)
	G3/GX	80 (69.6%)
Primary tumor size	T1 (<5 cm)	0 (0%)
	T2 (5–10 cm)	8 (7.0%)
	T3 (10–15 cm)	52 (25.2%)
	T4 (>15 cm)	19 (16.5%)
	unknown	36 (31.3%)
Histologic subtype	NOS	109 (94.8%)
	Epithelioid	2 (1.7%)
	Malignant Triton tumor	4 (3.5%)
NF1—related MPNST		23 (20.0%)
Distant metastases at diagnosis		10 (8.7%)
Resection of the primary tumor		97 (84.3%)
Unresectable primary tumor without metastases		11 (9.6%)
Metastases location at the start of the systemic treatment	Lung	72 (62.6%)
	Liver	6 (5.2%)
	Lymph nodes	14 (12.2%)
	Bones	7 (6.1%)
	Other	10 (8.7%)
	Primary tumor/local recurrence	58 (50.4%)
Previous (perioperative chemotherapy)		26 (22.6%)

**Table 2:** Number of cycles of therapy, objective response rate (ORR) and median progression-free survival (PFS) in the first line of treatment depending on the used chemotherapy regimen.

Scheme	Median Number of Cycles (range)	ORR % (*n*)	Median PFS (95% CI) [Months]	HR (95% CI)	*p*
ADIC (*n* = 47)	5 (1–11)	27.6% (13)	4.3 (2.3–6.3)	1	
HD-IFO (*n* = 40)	4 (1–13)	25.0% (8)	3.2 (2.7–3.9)	1.28 (0.83–1.98)	0.260
EI (*n* = 17)	4 (2–12)	23.5% (7)	4.6 (0–10.6)	0.97 (0.56–1.73)	0.961
AI (*n* = 6)	6 (3–9)	66.7% (4)	10.1 (4.3–20.6)	0.30 (0.09–0.96)	0.042
AP3 (*n* = 5)	8 (2–9)	0%	5.9 (5.6–6.3)	0.87 (0.34–2.20)	0.765

ADIC, doxorubicin + dacarbazine; AI, doxorubicin + ifosfamide; AP3, doxorubicin + cisplatin; EI, etoposide + ifosfamide; HD-IFO, high-dose ifosfamide.

**Table 3:** Median progression-free survival (PFS) in the first line of treatment depending on the used chemotherapy regimen in patients who received chemotherapy alone or chemotherapy with surgery/radiotherapy.

Scheme	*n* (%)	Median PFS (95% CI) [Months]	HR (95% CI)	*p*
Patients treated with chemotherapy alone (*n* = 82)				
ADIC	37 (45.1%)	3.6 (2.1–5.0)	1	
HD-IFO	25 (30.5%)	2.7 (1.3–4.1)	1.6 (0.95–2.70)	0.079
EI	13 (15.9%)	3.1 (1.1–5.1)	0.91 (0.48–1.72)	0.763
AI	2 (2.4%)	1.9 (NR-NR)	0.27 (0.04–2.00)	0.199
AP3	5 (6.1%)	5.9 (5.6–6.3)	0.76 (0.30–1.95)	0.565
Patients treated with chemotherapy and surgery/radiotherapy (*n* = 33)				
ADIC	10 (30.3%)	8.6 (7.7–9.4)	1	
HD-IFO	15 (45.5%)	6.5 (1.7–11.3)	1.31 (0.56–3.04)	0.534
EI	4 (12.1%)	7.5 (0.0–15.7)	1.36 (0.41–4.50)	0.616
AI	4 (12.1%)	12.1 (5.5–18.6)	0.48 (0.10–2.26)	0.353

ADIC, doxorubicin + dacarbazine; AI, doxorubicin + ifosfamide; AP3, doxorubicin + cisplatin; EI, etoposide + ifosfamide; HD-IFO, high-dose ifosfamide.

**Table 4:** Summary of most commonly used systemic therapies in the second, third, and fourth line of treatment.

Scheme	2nd Line (*n* = 74)		3rd Line (*n* = 40)		4th Line (*n* = 21)	
	*n* (%)	Median Number of Cycles (range)	*n* (%)	Median Number of Cycles (range)	*n* (%)	Median Number of Cycles (range)
Doxorubicin-based	50 (67.6%)	3 (1–12)	15 (37.5%)	3 (1–17)	5 (23.8%)	4 (1–12)
ADIC	35 (47.3%)	3 (1–11)	4 (10.0%)	2.5 (2–3)	2 (9.5%)	8.5 (4–9)
AI	0 (0%)	–	0 (0%)	-	1 (4.8%)	11 (11–11)
AP3	15 (20.03%)	3 (1–13)	11 (27.5%)	3 (1–17)	2 (9.5%)	1.5 (1–1)
Ifosfamide	2 (2.7%)	3 (1–4)	1 (2.5%)	3 (3–3)	0 (0%)	-
Etoposide-based	7 (9.5%)	4 (3–11)	8 (20.0%)	3 (2–16)	6 (28.6%)	4 (1–5)
Gemcitabine-based	9 (12.2%)	3 (2–11)	10 (25.0%)	3 (2–11)	4 (19.0%)	3 (1–8)
Pazopanib	1 (1.4%)	5 (5–5)	4 (10.0%)	3 (1–11)	4 (19.0%)	2.5 (2–4)
Other	5 (6.8%)	4 (2–8)	2 (5.0%)	2 (1–3)	2 (9.5%)	3.5 (2–5)

ADIC, doxorubicin + dacarbazine; AI, doxorubicin + ifosfamide; AP3, doxorubicin + cisplatin; doxorubicin-based regimens, doxorubicin alone or in combination with ifosfamide/dacarbazine/cisplatin; etoposide-based regimens, etoposide alone or in combination with ifosfamide/cisplatin; gemcitabine-based regimens, gemcitabine alone or in combination with docetaxel/paclitaxel.

## Appendix A

**Table A1:** Details of main chemotherapy regimens used in the treatment of patients with MPNST. ADIC, doxorubicin + dacarbazine; AI, doxorubicin + ifosfamide; AP3, doxorubicin + cisplatin; EI, etoposide + ifosfamide; GEM, gemcitabine; GEM+DXL, gemcitabine + docetaxel; HD-IFO, high-dose ifosfamide.

Regimen	Drug	Dose	Day (of 21-Day Cycle)
AI	Doxorubicin	25 mg/m^2^	1–3
	Ifosfamide	2.5 g/m^2^	1–4
ADIC	Doxorubicin	15 mg/m^2^	1–5
	Dacarbazine	150 mg/m^2^	1–5
AP3	Doxorubicin	20 mg/m^2^	1–3
	Cisplatin	30 mg/m^2^	1–3
HD-IFO	Ifosfamide	1.7 g/m^2^	1–7
EI	Etoposide	100 mg/m^2^	1–5
	Ifosfamide	1.5 g/m^2^	1–5
GEM	Gemcytabine	650 mg/m^2^	1, 8
GEM+DXL	Gemcytabine	650 mg/m^2^	1, 8
	Docetaxel	50 mg/m^2^	8
Pazopanib	Pazopanib	800 mg	daily

**Table A2:** Chemotherapy regimens used in the 1st line depending on the year of treatment start. ADIC, doxorubicin + dacarbazine; AI, doxorubicin + ifosfamide; AP3, doxorubicin + cisplatin; EI, etoposide + ifosfamide; HD-IFO, high-dose ifosfamide.

Scheme	Until 2005 (*n* = 35) *n* (%)	2006–2010 (*n* = 22) *n* (%)	2011–2015 (*n* = 44) *n* (%)	2016–2019 (*n* = 14) *n* (%)
ADIC	18 (51.4%)	5 (22.7%)	22 (50.0%)	2 (14.3%)
HD-IFO	16 (45.7%)	16 (72.7%)	8 (18.2%)	0 (0.0%)
EI	0 (0.0%)	0 (0.0%)	9 (20.5%)	8 (57.1%)
AI	0 (0.0%)	1 (4.5%)	2 (4.5%)	3 (21.4%)
AP3	1 (2.9%)	0 (0.0%)	3 (6.8%)	1 (7.1%)

**Table A3:** Progression-free survival (PFS) on different systemic therapy regiment irrespective of the line of treatment. HR, hazard ratio.

Scheme	PFS (95% CI) Months	HR (95% CI)	*p*
Doxorubicin-based	4.2 (2.8–5.9)	1 (ref.)	
Ifosfamide	3.2 (2.3–3.9)	1.29 (0.91–1.49)	0.153
Etoposide-based	3.1 (2.3–3.9)	1.04 (0.73–1.49)	0.834
Gemcitabine-based	2.4 (2.0–2.9)	1.74 (1.14–2.66)	0.010
Pazopanib	3.8 (1.8–5.8)	1.32 (0.74–2.34)	0.350
Other	2.1 (1.2–2.9)	1.90 (1.12–3.21)	0.017

ref., reference group for logistic regression model.

**Table A4:** Progression-free survival (PFS) on different systemic therapy regiment irrespective of the line of treatment. HR, hazard ratio in patients receiving chemotherapy alone.

Scheme	PFS (95% CI) Months	HR (95% CI)	*p*
Doxorubicin-based	3.1 (1.9–4.3)	1 (ref.)	0.06
Ifosfamide	2.5 (1.3–3.6)	1.48 (0.97–2.28)	0.072
Etoposide-based	3.0 (2.3–3.7)	0.85 (0.58–1.26)	0.418
Gemcitabine-based	1.7 (0.1–3.3)	1.83 (1.12–3.00)	0.016
Pazopanib	3.0 (1.7–4.4)	1.00 (0.57–1.91)	0.889
Other	2.1 (1.3–2.9)	1.31 (0.71–2.45)	0.383

ref., reference group for logistic regression model.
